# The quantitative paradigm and the nature of the human mind. The replication crisis as an epistemological crisis of quantitative psychology in view of the ontic nature of the psyche

**DOI:** 10.3389/fpsyg.2024.1390233

**Published:** 2024-09-12

**Authors:** Roland Mayrhofer, Isabel C. Büchner, Judit Hevesi

**Affiliations:** Department of Psychology, University of Regensburg, Regensburg, Germany

**Keywords:** replication crisis, quantitative psychology, human mind, epistemology, ontology

## Abstract

Many suggestions for dealing with the so-called replication crisis in psychology revolve around the idea that better and more complex statistical-mathematical tools or stricter procedures are required in order to obtain reliable findings and prevent cheating or publication biases. While these aspects may play an exacerbating role, we interpret the replication crisis primarily as an epistemological crisis in psychology caused by an inadequate fit between the ontic nature of the psyche and the quantitative approach. On the basis of the philosophers of science Karl Popper, Thomas Kuhn, and Imre Lakatos we suggest that the replication crisis is therefore a symptom of a fundamental problem in psychology, but at the same time it is also an opportunity to advance psychology as a science. In a first step, against the background of Popper’s Critical Rationalism, the replication crisis is interpreted as an opportunity to eliminate inaccurate theories from the pool of theories and to correct problematic developments. Continuing this line of thought, in an interpretation along the lines of Thomas Kuhn, the replication crisis might signify a model drift or even model crisis, thus possibly heralding a new paradigm in psychology. The reasons for this are located in the structure of academic psychology on the basis of Lakatos’s assumption about how sciences operate. Accordingly, one hard core that lies at the very basis of psychology may be found in the assumption that the human psyche can and is to be understood in quantitative terms. For this to be possible, the ontic structure of the psyche, i.e., its very nature, must also in some way be quantitatively constituted. Hence, the replication crisis suggests that the ontic structure of the psyche in some way (also) contains a non-quantitative dimension that can only be grasped incompletely or fragmentarily using quantitative research methods. Fluctuating and inconsistent results in psychology could therefore also be the expression of a mismatch between the ontic level of the object of investigation and the epistemic level of the investigation.

## 1 Introduction

Is the so-called replication crisis in psychology really a crisis that threatens psychology as an academic discipline in any way? Before answering this question, it is helpful to first outline the broader context. The replication crisis affects not only psychology, the focus of this study, but science as a whole, which is why important fundamental questions of philosophy of science are at stake here. The term “replication crisis” summarizes a number of problems that all revolve around the observation that certain results of scientific research cannot be replicated (for a summery, see Romero, 2019). Beginning in the 2010s, it was first noted for isolated, prominent topics—social priming as well as other findings from social psychology (Harris et al., 2013; Klein et al., 2014) and extrasensory perception (Galak et al., 2012)—then systematically across several areas of psychology that a substantial proportion of published studies, approximately between 23 and 62%, cannot be replicated or can only be replicated to a limited extent (Camerer et al., 2018; Klein et al., 2018; Open Science Collaboration, 2015). In other disciplines such as medicine (e.g., Ioannidis, 2005), economics (e.g., Camerer et al., 2016), natural sciences and engineering (e.g., Baker, 2016), it has also been found that only some of the published results can be replicated. Since replication of findings is a cornerstone of scientific methodology and the justification of knowledge, the term “replication *crisis*” was used for the observation that many findings cannot be replicated in order to express the notion that this is a—potential—problem (Romero, 2019).

At the same time, methodological problems have been intensively discussed in psychology since the 2000s, above all questionable research practices, i.e., practices that can be used to achieve significant results, from the exploitation of statistical aspects to make results significant, to non-transparent procedures to veil possible problems and present a found result as unambiguous, to the direct manipulation of data to achieve the desired result (for a summary, see O’Donohue et al., 2022). In psychology, the method—above all a quantitative-experimental approach—is generally predominant and confidence in theories is often greater than in the methods, so that the unexpected outcome of an experiment is often attributed to errors in the method, so that instead of modifying or discarding the theory, attempts are made to change the method so that the result predicted by the theory is achieved (Eronen and Bringmann, 2021; Mayrhofer and Hutmacher, 2020). This fundamental focus on methodology probably led to the replication crisis being viewed primarily as a crisis of methodology, in particular of the statistical methods used, and accordingly the solution would also lie in improved statistical methods. For example, the use of frequentist statistics, especially null hypothesis significance testing, was criticized and the increased use of descriptive (e.g., Trafimow and Marks, 2015) or Baysian statistics (e.g., Colling and Szűcs, 2021), a stronger focus on statistical power (e.g., Anderson and Maxwell, 2017; Shrout and Rodgers, 2018), effect sizes (e.g., Flora, 2020), confidence intervals (Amrhein et al., 2019), equivalence testing (Lakens et al., 2018), or reforming the use of the *p*-value (e.g., Anderson, 2020; Benjamin et al., 2018) were suggested as improvements. In addition, methods such as machine learning (Orrù et al., 2020), meta-analyses (e.g., Sharpe and Poets, 2020), structural equation modeling (e.g., Kline, 2023), multiverse (e.g., Oberauer and Lewandowsky, 2019) or speciation curve analyses (e.g., Steegen et al., 2016) were proposed as methods with which the replication crisis could be countered.

Besides these many proposals relating to statistical aspects—i.e., the way in which data is processed and interpreted numerically and mathematically—a second perspective aims at social-organizational aspects of the scientific process, namely proposals to prevent questionable research practices, to prevent publication bias or the file drawer problem, to mitigate the publish-or-perish problem, or to improve the institutional framework conditions of research in order to counter incentives for fraud (e.g., Asendorpf et al., 2013; Francis, 2012; Greenfield, 2017; Irvine, 2021; Koole and Lakens, 2012; Korbmacher et al., 2023; Lilienfeld, 2017). A third direction is aimed at the theories that underlie research (Fiedler, 2017 and 2018; Lilienfeld and Strother, 2020; Oberauer and Lewandowsky, 2019; Scheel et al., 2021; Scheel, 2022), but the focus there is on the fact that these proposals do not deal with individual specific theories and their content, but argue—on a meta-level, as it were—that generally better theories are needed.

Despite this extensive discussion revolving around the replication crisis and the many suggestions on how to counter the replication crisis, there is no evidence of specific negative institutional-systemic consequences, e.g., no psychological institutes at universities have been closed, and the performance and functioning of academic psychology has not declined either, in the sense that no less output in the form of articles has been produced than before the replication crisis. In fact, there is even evidence that non-replicable studies are cited more often than replicable ones (Serra-Garcia and Gneezy, 2021). From this perspective, then, it appears that the failure to replicate certain findings has had little or even no impact on psychology as an academic discipline. There are also voices that argue that the observation that results cannot be replicated is not problematic at all (Haig, 2022; Maxwell et al., 2015; Schmidt and Oh, 2016; Stroebe and Strack, 2014). Yet this perceived need to defend the status quo and counter ideas of a crisis in itself and, conversely, the many suggestions on how to counter the replication crisis, suggest that there is an important and fundamental issue at stake here. The present study argues, first, that at its core the replication crisis is not a methodological or social-institutional crisis, but rather—following a suggestion by Morawski (2019)—an epistemological crisis revolving around the question of how to justify the knowledge that psychology generates. Second, while what has been called the replication crisis is indeed a substantial problem for psychology, this crisis also opens up the possibility of clarifying fundamental epistemic and ontic questions in psychology. The ontic implications associated with this epistemological crisis are also discussed, i.e., whether the core of the replication crisis—and in a broader sense of psychology as a scientific discipline—is to be found in the very nature of the psyche itself, and whether the research methods used are not or only partially capable of grasping this nature.

Three classics of philosophy of science—Karl Popper, Thomas Kuhn, and Imre Lakatos—provide a promising framework for analyzing the replication crisis from a philosophy of science perspective. Although these theoretical approaches focus on different aspects and are also considered incompatible in some cases, together they can offer explanations that make the replication crisis more comprehensible, as will be shown below. The focus here is primarily on epistemological aspects, and accordingly the replication crisis is viewed here primarily as an epistemological crisis and less as a methodological crisis, more precisely as a consequence of an inadequate approach to the human mind as the object of investigation in psychology. The replication crisis, as well as many proposals on how to deal with it, are very much focused on quantitative aspects, namely the quality of data and its statistical analysis, but at the same time it remains doubtful whether these proposals have led to improved replicability. Therefore, this study proposes the possibility that the human psyche—possibly due to its very nature—at least partially resists access through a quantitative perspective and approach, of which the replication crisis may be a symptom. Therefore, if the epistemological approach to the psyche through a primarily quantitative perspective does not fit the fundamental ontic structure of the psyche, it is to be expected that the corresponding results are ambiguous and instead point to a fundamental problem, i.e., that an epistemological crisis occurs.

## 2 Perspectives from philosophy of science and their consequences for psychology

### 2.1 The Popperian perspective: failed replication as the opportunity to improve theories

According to Popper’s (1959/2005) Critical Rationalism, a perspective on science which is widespread in academic psychology, the failure to replicate certain findings is part of the “normal” and even desirable progress in science (see also Derksen, 2019; Laws, 2016; Keuth, 2005; Rowbottom, 2011). Falsified hypotheses are rejected and hypotheses that have withstood attempts at falsification are retained—at least for the time being, and at least according to Popper’s idea of ideal science. Many proposals concerning the replication crisis accept the basic epistemological premise, largely based on Fisher’s (1935/1974) and Popper’s (1959/2005) influential books, which have substantially shaped the methodology and the self-conception of psychology, that reproducibility is one of the basic requirements of science in order for its results to be justifiably considered knowledge. Popper started from the so-called problem of induction, i.e., the question of whether and how inductive conclusions can be justified. On the one hand, a large number of similar observations allow the prediction that the same phenomenon will also be repeated in the future, but on the other hand, recourse to past observations cannot guarantee that this will also apply to the future. Popper “solved” the problem of induction—a more detailed analysis of this intricate problem lies outside the scope of this article (see, e.g., Agassi, 2014; Musgrave, 1993; Swann, 1988)—by reversing the problem, so to speak, and postulating instead that theories should not be verified but rather falsified. Therefore, replications, which in principle are the repetition of an observation, play an epistemologically subordinate role because they “only” confirm, i.e., “verify,” previous observations, and according to Popper verification is impossible in principle. Verifications do support theories, and theories that are supported by many observations—or, according to another interpretation, that have withstood many attempts to disprove them—are considered more likely to be true, but theories cannot be proven by repeated identical observations, only be disproved by conflicting observations.

This raises the question of how to interpret a replication: Is it an attempt at verification that adds another confirming observation to a theory if the replication is successful, thus increasing its probability of being true? And if so, how many successful replications are necessary for a theory to be accepted as true with some probability? In other words, can knowledge be quantitatively justified? Conversely, is an unsuccessful replication attempt—perhaps even a single unsuccessful replication—to be equated with a refutation of the theory in question? Or is an unsuccessful replication merely the lack of confirmation of a theory that, according to Popper, has a lower epistemological value than a direct refutation? Although the answer may depend on the specific theory in question, clarifying these questions is crucial to understanding the replication crisis and its epistemological dimensions.

It is also necessary to clarify what exactly is meant by replication. In psychology, people—and not inanimate matter—are usually studied, and therefore a completely exact replication is impossible in almost all cases because study participants are changed by their very participation, so that a study cannot be conducted with the same people and new participants necessarily differ from the previous ones. Epistemologically, it could be argued that people often differ only slightly, at least in a particular aspect which is of interest in a study, that said aspect is distributed in a certain way, which allows a statistical approach, or that with a sufficiently large sample the mean can be used as an estimator, and it is therefore justified to speak of replication as long as the study design itself remains unchanged. Interestingly, all of these points contain a more or less clear quantitative component: This is evident in statistical aspects, but statements about the size of differences also imply at least a rudimentary quantitative understanding. This is a first indication that the human mind—at least in certain aspects—is regarded as quantitatively constituted in psychology and thus meaningfully accessible to quantitative methods.

However, even if one accepts these arguments concerning replication, the question arises as to the time periods for which such equality is assumed, as cultures and societies, and therefore also people, change over time—and change to such an extent that psychological processes may also be affected (e.g., Hutmacher and Mayrhofer, 2023). This problem obviously exists with standardization and calibration, for example with intelligence or personality tests that have to be updated over time, or with test–retest reliability in general, so that the question arises as to whether other psychological processes—e.g., cognition, motivation, or emotion—also change over time. On a more practical level, exact replications also appear difficult, as they may be carried out by other investigators, in translation, with different materials, or in other cultures, all of which may influence the outcome. This is illustrated by the well-known WEIRD bias in psychology, according to which the majority of the results of psychological research are obtained from a very specific group, namely American undergraduates, that is hardly representative of humanity as a whole, but the results are often regarded as universally valid (Henrich et al., 2010). Thus, from how much deviation do we no longer speak of replication? Even this brief sketch shows that the question of the basic conditions for replication is not easy to answer.

From a different perspective, however, another problem can be identified here that is even more fundamental in terms of epistemology. If replications are suitable for supporting or refuting the validity of theories, then this presupposes that the way in which the associated empirical observations are carried out and measured is also suitable for answering the theory or research question in a meaningful way. Otherwise, neither corroboration nor refutation would be possible, because the measurements, data, and results as well as the conclusions drawn from them would have no meaning then and could not be interpreted as corroboration or refutation either.

Now, all studies that were examined and replicated for the original replication project (Open Science Collaboration, 2015) and its continuation (Camerer et al., 2018) were experimental psychological studies in which a quantitative methodology was used. This fact in itself is remarkable, because these experiments were intended to be representative of (experimental) psychology (Open Science Collaboration, 2015) or they appeared in prestigious journals (Camerer et al., 2018). Furthermore, the experiments were also chosen for practical reasons, namely, that “[t]he key result had to be represented as a single statistical inference test or an effect size” (Open Science Collaboration, 2015 p. 2) or that there was a “clear hypothesis with a statistically significant finding” (Camerer et al., 2018, p. 1). The analysis of the replications carried out and the subsequent interpretation that many previous findings could not be replicated was also quantitative. Since it is difficult to specify clear quantitative criteria for when a replication is successful or not (e.g., Chambers, 2017; Cumming 2008; Gelman and Stern, 2006; Open Science Collaboration, 2012, 2015; Simonsohn, 2015; Verhagen and Wagenmakers, 2014), the problem described above of how replications are to be classified in terms of epistemology theory is further exacerbated.

Although it remains to be discussed whether an unsuccessful replication represents a refutation, the failure to replicate findings is critical in Popper’s view as the theories in question are not corroborated and thus prone to rejection and elimination from our pool of theories, being replaced by theories that are better supported by repeated observations. From this perspective, the replication crisis is not a crisis at all but rather a process that increases our knowledge by demonstrating that certain theories are false or at least cannot be corroborated by repeated observations, increasing their probability of being false. Therefore, notwithstanding the many problems of the various forms of Critical Rationalism (e.g., Agassi, 2014; Keuth, 2005; Rowbottom, 2011), the Popperian perspective offers a different view on the replication crisis: From this point of view, the replication crisis can be seen as a corrective pruning process because it allows the discovery of potentially false theories, which can be removed from our pool of theories, thus creating space for new theories that are closer to truth.

### 2.2 The Kuhnian perspective: unexpected observations as a harbinger of a model crisis

Karl Popper and Critical Rationalism assume that there is an objective truth and, based on this, that knowledge is also objective. In contrast, Kuhn (1966; see also Marcum, 2005; Nickles, 2003) strongly emphasizes the social dimension of science as a collective process. In a nutshell, Kuhn assumes that science is not a more or less linear process in which we get steadily closer to truth over time. Instead, a cyclical model is postulated in which different paradigms^1^ replace each other. Once a paradigm has established itself and is considered to be true, further research then takes place within this paradigm—the so-called “normal science.” This is not only a purely “rational” process, in which exclusively only aspects that are directly related to the object of knowledge are decisive, but also other, mainly social, factors play a role in which this is not the case and which instead indirectly affect a paradigm, e.g., influential persons who control the flow of resources or the allocation of academic positions and who can therefore influence other researchers, or general cultural and social conditions that favor thinking in a certain direction and marginalize other directions. However, at some point the first observations are made that do not appear compatible with the prevailing paradigm—the first signs of a so-called “model drift.” Initially, these observations are simply ignored or labeled as anomalies, but over time there is mounting evidence that the prevailing paradigm does not represent the (whole) truth—what is called “model crisis.” Eventually, the prevailing paradigm can no longer be maintained and a “model revolution” occurs in which a new paradigm prevails, which then becomes the new normal science. In this process, it must be taken into account that not only “rational” factors directly related to the object of knowledge play a role, but also—as already mentioned—social or cultural factors, such as when influential persons who upheld a paradigm no longer (can) perform this function.

According to Kuhn’s model, which is less epistemologically and more sociologically oriented, crises that give rise to doubts about previous knowledge are processes that occur regularly and more or less systematically. From a formal point of view, i.e., if the cycle described above is regarded as a theory that can describe and predict the course of science, it may be assumed that the replication crisis could signify a model drift or even a model crisis as unexpected observations have emerged.

These observations are unexpected because, according to the current state of knowledge—i.e., high-ranking published studies in which a specific psychical^2^ phenomenon is described—it should be assumed that this knowledge is reliable and can therefore be replicated by and large. There are three possible reasons why this is not or only partially the case: first, the original knowledge, i.e., the original studies, is false, so the failed replications are correct. Second, the original studies describe true phenomena and theories but the replications are—for whatever reason—untrue. These two possibilities could presumably be clarified by carrying out many replications, perhaps also with additional variations, in order to be able to determine the influence of different effects and variants (e.g., Breznau et al., 2022; Muñoz and Young, 2018; Silberzahn et al., 2018; Simonsohn et al., 2020; Steegen et al., 2016; Young, 2018). If there are clear tendencies, it would be possible to recognize whether the effect or mechanism postulated in the original study actually exists in a general form or whether it is merely an individual situation that resulted from certain idiosyncrasies. Therefore, these possibilities can be dealt with within the currently prevailing paradigm, i.e., the so-called normal science.

A third possibility, however, is that it is not possible to say with any certainty whether the original study or the replication is true. This possibility can be attributed to the assumption—as explained later in this study—that both the original studies and replications may not be suitable for adequately grasping the psychical phenomenon of interest. Such an inadequate fit between phenomenon and research method leads most likely to inexplicable results in the observation and analysis of the phenomenon, which cannot be understood within the paradigm of normal science because the theoretical and conceptual foundations are not sufficient. This connection was demonstrated by Kuhn (1966) and Feyerabend (1975), primarily using examples from astronomy, and even if the controversial question of whether a general theory of how science works can be derived from this is excluded (e.g., Farrell, 2003; Oberheim, 2006; Preston, 1997), these cases illustrate the possibility of a model crisis and a paradigm shift.

For psychology and the replication crisis, it is now relevant that the methods used reflect the paradigm within which they are used. Therefore, unexplained results may indicate that the interplay of basic theoretical assumptions and methods is not appropriate to the phenomenon under investigation, casting doubt on the underlying paradigm, thus possibly heralding a model crisis or even model shift in psychology.

So, while Kuhn’s theory can explain the systemic and social reasons why a paradigm shift occurs in science, it does not, in terms of the specific scientific content, provide explanations as to why the “anomalies” challenge the prevailing paradigm. While this complex fundamental question (e.g., Fuller, 2003; Lakatos and Musgrave, 1970; Toulmin, 1972) lies outside the scope of this analysis, the model of paradigm shifts nevertheless seems to imply that some theories somehow fit empirical observations better than others. Abstractly speaking, Kuhn’s model thus always contains an epistemological crisis, and since—as shown above—the replication crisis is an epistemological crisis, it can consequently be interpreted in Kuhnian terms as a model crisis or even model drift. Furthermore, merging the more specific epistemological level, as described above in Popper’s model, with Kuhn’s model, justification of knowledge plays an important role in both cases because, epistemologically, failed replications can lead to an undermining of existing knowledge, which in turn anticipates a model crisis and, eventually, a model revolution and paradigm shift.

Furthermore, Kuhn’s model may be supplemented by the observation that over time models and procedures can lose their connection to the actual object of investigation and instead only revolve around themselves (Elster, 2016), meaning that in the last phases before a paradigm shift, the traditional way of doing science—“normal science,” in Kuhnian terms—loses its vitality and fossilizes. Interestingly, when this happens, there can also be a tendency toward “mathematical sophistry,” so that the methodological tools also lose their relation to the phenomena being investigated and instead become a purposeless “toy” (Elster, 2016, p. 2182).

### 2.3 The Lakatosian perspective: the role of methodology in psychology

Lakatos’s (1978; see also Larvor, 1998) philosophy of science focuses on the concept of the so-called “research program.” This is a central set—called the “hard core”—of related, interdependent axioms, concepts, theories, and possibly also methodologies, which provide the foundations, guidelines, and directions for research and that cannot be abandoned or altered without compromising the research program itself. Around the hard core, there is a protective belt of so-called auxiliary hypotheses, which usually concern methodological aspects and deal with anomalies or observations contradicting or inconsistent with the central assumptions of the hard core. Rather than disputing the hard core itself, which would challenge the very foundations of the research program, problems that arise from such conflicting observations—in Kuhnian terms, the “anomalies”—are rerouted to the protective belt. Thus, instead of modifying or abandoning the central assumptions of the hard core, attempts are first made to defuse “problematic” observations by dealing with them at the level of auxiliary hypotheses, i.e., usually at the methodological level, trying to explain said observations by methodological errors, inaccuracies, or other shortcomings. If this is not or no longer possible, the auxiliary hypotheses can be modified so that “problematic” observations can be explained without compromising the hard core.

There are, however, two crucial points: First, the auxiliary hypotheses and the protective belt must somehow be conceptually related to the hard core, i.e., the auxiliary hypotheses and the protective belt must not be incompatible with the hard core because otherwise they could not protect the hard core at all but would rather challenge it. Second, the line between fundamental concepts and the hard core and auxiliary hypotheses and the protective belt is not always clear-cut. This makes it difficult to decide if modifications affect only the auxiliary hypotheses, i.e., if the protective belt functions actually as protection of the hard core or if the ramifications are so far-reaching and profound, going beyond the protective belt, that the hard core itself is affected by assumptions that were originally meant to protect it. Accordingly, the hard core is only abandoned if conflicting data and contradictions can no longer be rerouted to and resolved within the protective belt.

Complicating matters further, the extent of a hard core is a matter of discussion. In the case of psychology, there is no clear hard core as focal point for the whole discipline or its branches because the subject matter, namely human mind and behavior, is very vast and diverse and there is presently no fundamental or all-encompassing theory which might provide a coherent framework for a research program in the Lakatosian sense. For much of the 20th century, behaviorism can be regarded as research program because the fundamental idea that virtually all behavior can be explained in terms of stimulus, response, and contingencies provides a coherent and all-encompassing theory as the basis for a research program. Evolutionary psychology and behavioral neuroscience may be regarded as attempts to establish a hard core in the Lakatosian sense for psychology, because both operate from the basis of a single fundamental theory, namely that mind and behavior can be explained by evolutionary or biological processes, respectively. However, none of these approaches has gained near-universal acceptance or has produced decisive results to dominate academic psychology.

On a less global level, certain paradigms could be seen as research programs, such as the idea in neuropsychology that certain behaviors, personality traits, or mental disorders can be localized in certain places in the brain (e.g., Corr et al., 2013; Dolan and Park, 2002; Shenal et al., 2003; Schretlen et al., 2010). In cognitive psychology, the testing effect can be interpreted as a research program because, built on a fundamental assumption, namely the effect of retrieval, further theories are grouped together (e.g., Rowland, 2014; Schwieren et al., 2017) which—and this is the crucial point—would immediately lose their validity if the effect of retrieval as a common focal point would turn out to be false.

Despite the lack of a hard core of fundamental and universal theories in contemporary psychology, there nevertheless seems to be some kind of unifying factor which provides coherence to psychology as an academic discipline, namely the focus on a methodology that is characterized by experimental, quantitative, and empirical approaches (Mayrhofer and Hutmacher, 2020). This observation is crucial for any analysis in Lakatosian terms because it can be argued, on the one hand, that the dominance of this methodology constitutes a research program by providing a coherent frame within which research in psychology is conducted. On the other hand, the hard core of a Lakatosian research program is not—at least not primarily—characterized by a certain methodology *per se* but rather by central concepts and theories, and the preferred or characteristic methodology reflects the supposedly best way to investigate the central concepts and theories.

Therefore, it seems that the quantitative-experimental methodology fulfills a dual role: First, it acts as a “protective” belt of auxiliary hypotheses that virtually defines how psychical phenomena are approached, thus shielding the core from questions or problems which cannot be approached quantitatively, empirically, or—to a lesser extent—experimentally. Consequently, psychological phenomena that are not accessible to such a quantitative-experimental approach are sidelined and eclipsed by the vast research conducted according to those very principles. Second, at the same time, there is no fundamental universal theory that could explain all these phenomena and thus serve as the focal point and hard core of a research program. Since such a blank space cannot hold together a research program, methodology takes on this task as a substitute, as it were. Taken to its logical conclusion, this means that the methodology protects itself—which is a somewhat paradoxical statement that will be explained in more detail below.

However, while it remains unclear what the hard core actually is, the shielding function of the protective belt can also be analyzed from the question of whether a research program is—in Lakatosian terms—progressive or degenerative (Lakatos, 1978). Modifications in the auxiliary hypotheses can prompt further advancements and refinements within the research program, thus strengthening the hard core and the fundamental theories by clarifying problems or correcting minor errors and defects in the central concepts and theories. In this case, the research program is considered progressive because it produces new knowledge and its explanatory power is increased. If, by contrast, modifications in the auxiliary hypotheses do not improve the hard core but simply serve to shield it from conflicting observations, thus actually decreasing the scope and explanatory power of the fundamental theory, the research program is considered degenerative.

Lakatos (1978) discussed the relationship between methodology and the hard core of the fundamental theories in terms of the so-called positive and negative heuristic. Based on a more differentiated interpretation of modus tollens than in Critical Rationalism, the negative heuristic states that observations inconsistent with the fundamental theories should not be immediately regarded as falsifications, thus protecting the hard core. The consequence is that discussions about how challenging observations should be interpreted and handled often take place at the level of the auxiliary hypotheses, i.e., in the protective belt, which comprises the methodology as well. The positive heuristic, on the other hand, acts as a methodological framework within which research is carried out. It provides certain strategies, tools, and techniques to solve problems and answer questions that are typical for the research program. Successful approaches yielding fruitful results usually become the methods of choice precisely because they have shown their efficacy and thus promise to be able to answer further questions as well. As a consequence, however, relying on a “tested” and “safe” methodology also implies or even determines what kind of problems and questions are addressed—namely those compatible with the preferred methodology.

Against the background of Lakatos’ theory, the replication crisis can be interpreted as follows. According to Lakatos, if a substantial number of findings cannot be replicated—i.e., anomalies occur, in Kuhnian terminology—this problem is first dealt with at the level of the protective belt. This assumption fits with the observation that the discussion on the replication crisis primarily revolves around the level of methods, i.e., improving data quality and analysis. This discussion takes place on the level of the protective belt, because being about methodology it is about access to psychical phenomena and not about the psychical phenomena themselves. Therefore, this discussion reflects a fundamental epistemological problem, namely the question of how to gain appropriate access to psychical phenomena, i.e., the object of investigation in psychology.

However, since—as explained above—it remains unclear and vague what exactly the hard core of psychology consists of and instead the methodology, i.e., a quantitative approach, vicariously assumes the role of giving the discipline a structure and the research activities a direction in the sense of a Lakatosian research program. However, if the methodology of psychology is called into question, it is not only the protective belt that is affected, but also the very core. Due to this peculiarity, fractures in the protective belt thus also affect the core of psychology, and these potentially far-reaching consequences point to a model crisis in the Kuhnian sense.

### 2.4 The quantitative paradigm and the replication crisis

The questions of whether a research program—in Lakatos’ sense—is progressive or degenerative, and whether a positive or negative heuristic is present, can be applied to the replication crisis. Many suggestions on how to counter the replication crisis revolve around the improvement of statistical methods, i.e., quantitative methods. Against the background outlined above, this is important in several respects:

First, this discussion can be interpreted as a typical methodological discussion that takes place at the level of the auxiliary hypotheses, precisely because the methods of a research program are the focus and not the underlying theories of psychical phenomena themselves. Second, the discussion about means to solve the problems raised by the replication crisis is characterized by ambivalence: On the one hand, if these proposals are successful, these changes in methodology, i.e., at the level of the auxiliary hypotheses, would improve the ability of the hard core to deal with problematic observations, which would be progressive. On the other hand, it is doubtful whether the elimination of a problem—lack of replicability—can actually be seen as generating new knowledge and increasing the explanatory power of the theories of the hard core. From this perspective, it would therefore be more appropriate to speak of a defensive discussion that attempts to solve problems by eliminating anomalies, which would qualify the research program as degenerative.

Third, this is all complicated by the fact that it is unclear what the hard core actually is and what its basic assumptions and theories are. However, if a large part of the discussion on how to counter the replication crisis revolves around methodological questions, and if these methodological questions are discussed independently of the content of psychical theories, the auxiliary hypotheses in the protective belt do not protect the hard core of psychical theories but rather the methodology itself. Improving the methodology without tying it to genuine psychical theories is epistemologically problematic because then the methodology revolves around itself and the research program becomes degenerative.

Viewed more generally from a philosophy of science perspective, a mismatch between methodology and psychical theories can also be interpreted as an insufficient or inadequate understanding of the ontic nature of the object of investigation—in this case the psyche—from which a set of fundamental interrelated epistemic problems arises. Although the object of study in psychology is obviously the psyche, a precise definition of this term is difficult and controversial (e.g., Mayrhofer and Hutmacher, 2020). This difficulty in finding a common denominator for cognitive, emotional, motivational phenomena and the like is a first indication that a fundamental issue is at stake here. For the purposes of this study, however, it is sufficient to understand “psyche” unspecifically—and somewhat tautologically—as the totality of psychical phenomena as studied by psychology. The ontic nature of the psyche refers to the fundamental being or essence—in a philosophical sense—of the psyche itself and not how it functions. Classical concrete ontic questions, such as the conditions of the possibility of being (here: of the psyche) in the abstract sense but also the mind–body problem (Weir, 2024) or questions about the nature of consciousness (Rowlands, 2001) or emotions (Soteriou, 2018) can be largely excluded here, because the focus is on the abstract relationship to the epistemic level.

The aim of ontology (e.g., Effingham, 2013) is not only to understand the nature of being and what it means for something to exist (in a certain form), but also to categorize (ontic) entities, to clarify their relationship to each other and the principles governing their functioning. By addressing the most fundamental ontic aspects of an object (of investigation), ontology also provides a frame of reference for other disciplines by clarifying the fundamental structures and conditions that constitute the object of investigation. Epistemology deals, in short, with everything that has to do with the nature of knowledge, its generation and justification (e.g., Carter and Littlejohn, 2021). What we know and can know about an object is therefore not only an epistemic question—e.g., which methods can be used to approach the object, to what extent the object is recognizable at all, or how the object can function in principle—because the answers to these questions are obviously (also) enabled, determined and limited by the ontic nature of the object. Thus, the ontic structure of an object necessarily affects our epistemic understanding of it and knowledge results if the ontic and epistemic levels are in agreement (Bachelard, 1974; Sandkühler, 1991). For the way in which such an object is constituted in terms of its ontic structure also determines the possibilities of grasping it epistemically. One of the reasons why such an investigation is possible is that the investigating entity, i.e., humans, must somehow—and the exact nature of this relationship is disputed—be compatible with the object of investigation due to its own ontic constitution, because otherwise the investigating entity would have no way of understanding the object of investigation. The ontic relationship between the object of investigation and the investigating entity thus determines the epistemic possibilities of the investigating entity to grasp and understand the object of investigation (for a summary, e.g., Jacquette, 2014; Morawski, 2019; Steup, 1996; Steup and Ram, 2024).

However, if the epistemic and ontic levels are mismatched far-reaching and serious problems can arise, for example if assumptions are made on the epistemic level about how to approach the object of investigation that do not match the ontic structure of the object of investigation, are incompatible with it, or even contradict it. First, the object of investigation and how things work cannot be understood, or can only be understood inadequately, or in a distorted way. Second, as a direct consequence, the unreliable knowledge thus produced and obtained is not suitable as a basis for making correct predictions, interventions, and manipulations, as this knowledge reflects reality only inadequately, distortedly, or even falsely. Thus, the mismatch between the knowledge produced and experiences in reality becomes evident. Third, this results in problems in justifying the knowledge produced in this way—even if it is partially correct and reliable—because it is not systematically correct, but at best selective and possibly for unclear, random reasons. This means, fourth, that a scientific discipline is thus likely to produce anomalies and enter into a crisis (in Kuhnian terms) or to stagnate or degenerate as a research program (in Lakatosian terms).

If the replication crisis, as argued above, is indicative of a fundamental epistemic problem in psychology, this problem could lie precisely in such a mismatch between the epistemic and ontic levels. In concrete terms, this means that a fundamental aspect or dimension of the ontic nature of the psyche may not be understood, insufficiently understood, or misunderstood and thus neglected or inadequately addressed in research. As this dimension is not considered in research, but— presumably— is nevertheless present and affects the functioning of the psyche, research and its results are influenced by this unknown and unconsidered factor, which in turn could explain the anomalies and fluctuating results seen in the replication crisis. In other words, the replication crisis may be interpreted as an epistemological crisis rooted in an inadequate understanding of the ontic constitution of the psyche, leading to a mismatch between methodology and the epistemic level on the one hand, and the nature of the psyche as an object of inquiry on the other.

### 2.5 Psychology and the nature of the human mind

Considering the highly quantitative nature of psychology as a whole, as well as the proposed solutions to the replication crisis, which very often focus on quantitative aspects, this could be an indication that the root of this mismatch lies precisely here. This means that the human psyche might not be or only partially be accessible to investigation by quantitative methods—or theories based on quantitative thinking—due to its very ontic constitution. As a consequence, improvements in quantitative methods cannot resolve or mitigate the problems of the replication crisis.

That the replication crisis is a symptom of a fundamental problem in psychology, and that it revolves primarily around a methodology that is by its nature primarily quantitative, thus suggests that the mismatch between the ontic and epistemic levels may be rooted precisely in the quantitative nature of the methodology. This is because adequate access to the object of investigation using quantitative methods presupposes that it can also be grasped quantitatively. If problems arise, it is possible that the object of investigation cannot be grasped quantitatively because its ontic structure is such that certain aspects somehow elude such quantitative access. This suggests that the psyche contains a non-quantitative dimension, meaning the following: Ontological categories are an extensive and complex fundamental topic of philosophy on which there is little agreement (Perović, 2024; Westerhoff, 2005). Although quantity—i.e., how many?—has been considered a fundamental ontological category since Aristotle, what matters here is not what quantity the psyche—or its subsystems and mechanisms—has, but rather that it is quantitatively accessible at all. In order to be quantitatively accessible, the psyche must possess the ontic property of quantitativeness—to be quantitative—that is, to be composed and accessible in quantitative form and to be expressible and conceivable in quantitative, numerical terms. This does not mean that (latent) constructs such as intelligence or certain personality traits are represented in quantitative-numerical terms—and the difficulties in this endeavor are possibly another indication that the psyche contains a non-quantitative dimension—because this is merely an attempt to grasp something quantitatively at the epistemic level. And this attempt does not necessarily guarantee that intelligence or personality traits—apart from their controversial ontic status anyway—actually *are* quantitative in their ontic nature *eo ipso*. The same applies to attempts to grasp and understand subjective experience, aesthetic perception, dreams, unconscious processes and the like through psychological research. This problem is further exacerbated by the fact that there is no consensus on what the nature of the psyche actually is, as illustrated by the multitude of different ideas ranging from Plato’s concept of a tripartite soul to current neuroscientific concepts. Interestingly, these concepts do not take into account the question of a possible quantitative dimension of the psyche. For concepts prior to, say, the 19th century—i.e., more or less the beginning of psychology as a science in the modern sense—this is hardly surprising, since, generally speaking, until that time there was little or at least much less thinking in quantitative terms. However, for more modern concepts, which are based more on thinking in quantitative terms, as is typical of modern science, it is quite surprising if such a fundamental question was or is not explicitly discussed, but rather—more or less implicitly—assumed. Although modern ideas of the psyche, such as in psychometrics, behavioral economics, or neuroscience, work with quantitative methods, there has hardly been any discussion to date as to whether this also implies that the psyche is also—in whatever form—quantitatively constituted.

The question of how such a possible non-quantitative dimension of the psyche is to be understood lies beyond the scope of the present study for two reasons: First, answering this question requires extensive research, and second, the aim of this study is to explore quantitativeness as a possible ontic category of the psyche from a philosophy of science perspective and to elaborate the implications for psychology as a scientific discipline. Quantitativeness as a possible ontic category of the psyche, and in particular the property of “non-quantitative” as an explanation for difficulties such as those made visible by the replication crisis, is therefore primarily a matter of identifying a fundamental philosophy of science problem of psychology as a scientific discipline and making it recognizable as a problem. A more precise definition of this problem, describing its specific characteristics and then developing possible solutions are steps that necessarily follow.

Thus, this study raises the possibility that the ontic structure of the human psyche contains a dimension that is not quantitatively constituted and therefore to a certain extent eludes quantitative access. This does not mean that a phenomenon such as intelligence or a cognitive mechanism cannot be approached quantitatively in some form—in the case of intelligence this actually works quite well—but there is always the possibility that decisive aspects are not covered, which can lead to inexplicable variance, as exemplified in the replication crisis. In other words, it is possible that an epistemological crisis can be traced back to an insufficient epistemic fit with the underlying ontic structure, which possibly contains a non-quantitative dimension that could explain that insufficient fit. The nature or ontic structure of this something—be it directly intelligence or personality itself or a currently unknown underlying phenomenon—is relevant in this context, since it is the ontic structure that provides the basis for the phenomenon to be epistemically accessible and comprehensible. The same applies to cognitive, motivational, or emotional mechanisms as well as to consciousness, all of which can be observed—as surface phenomena, so to speak—but whose ontic structure is still completely unclear.

Three examples can be used to illustrate, at least to some extent, what such a non-quantitative dimension might look like: First, questions about qualia (e.g., Nagel, 1974; Tye, 2021) or meaning (e.g., Flanagan, 2007), which are fundamental to human psychical experience, have so far eluded not only any quantitative approach, but also a precise determination of their ontic nature. Second, the same applies to language, which in principle cannot be quantified either, because it works with meanings (e.g., Lycan, 2019; Platts, 1997). Third, Jaeger et al. (2024) have argued that agency, cognition, and consciousness cannot be computational or formalized or captured by algorithmic approaches. These examples thus suggest that a non-quantitative dimension exists in the ontic structure of the human psyche, even if it cannot yet be described in more detail.

The question of the ontic structure and nature are closely related to another—unsolved!—fundamental ontic problem of psychology, namely the mind–body-problem. Quantitativeness as an ontological category and the assumption of a non-quantitative dimension of the human psyche is in principle compatible with all three fundamental positions: In idealistic positions, a non-quantitative dimension must be thought of as immaterial, which in turn raises the question of what this looks like in concrete terms. With materialistic positions, the additional question arises as to how a non-quantitative—or quantitative, for that matter—dimension can be derived from a material basis. Dualistic positions are faced with the problem of which side—or possibly both?—quantitativeness is associated with, whether it manifests itself differently in each case, and what the interaction looks like in concrete terms.

## 3 Discussion

Mathematics is magic, literally and metaphorically. Literally, because magic attempts to depict the world in some form using abstract symbols and to change that what they represent by manipulating these symbols. In mathematics, concrete things or relationships are also represented in abstract form, namely by numbers and mathematical operations, and the manipulation of this representation makes it possible to make actual changes in the world—and this very often works. And is it not, metaphorically speaking, “magical”—in the sense of astonishing, because this connection is currently neither ontically nor epistemically fully explicable (e.g., Crump, 1992; Horsten, 2023; Shapiro, 2000)—that complex facts of the concrete, material world can be expressed, via universal laws, in abstract and seemingly unambiguous form as numbers and that the manipulation of these numbers can in turn influence the material world?

Against the background that the ontic status of numbers and mathematical operations is still as unclear as their epistemic possibilities and limits, the question arises in a discipline such as psychology, which relies very heavily on quantitative methods, whether there are limits to the use of quantitative methods, where these limits might lie, and what this means for psychology in general as an academic discipline.

Before discussing the implications of this assumption below, it should be noted that the present study is not intended to be prescriptive and no statements are made here about how psychology or, more generally, science should operate. Such claims, as advocated by Critical Rationalism or Logical Empiricism, are now regarded as outdated by philosophy of science and inappropriate for a complex endeavor such as science (e.g., Bird, 2013). Instead, the aim of this study is to identify and discuss possibilities concerning a fundamental problem, i.e., to explore what aspects that have been less or not yet addressed could also be relevant for psychology. Furthermore, it should be noted that science, and thus also psychology, is extremely complex, so that considerations of a general nature, such as those made here, necessarily only represent a rough and abstract outline.

The question of whether the human psyche is non-quantitative or contains a non-quantitative dimension in addition to a quantitative dimension is obviously extremely complex and extensive and goes far beyond the scope of the present study. Moreover, the term “non-quantitative” initially only represents a negative demarcation and an antithesis to the idea that the psyche is exclusively or primarily quantitative. The term “non-quantitative” is not intended at this point to provide a more detailed definition of what such a non-quantitative dimension might look like in concrete terms. On the one hand, this would have to be the subject of a comprehensive discussion from the perspective of various disciplines, which obviously goes far beyond a single study. On the other hand, it is equally unclear what is actually meant by “quantitative”—as a quantitative dimension of the psyche—and what it might actually look like if the psyche functions in a quantitative way. Approaching and possibly clarifying this problem would not only shed light on a fundamental question, but would also put psychology as a discipline on a better footing, as it can be assumed that such knowledge would also change our understanding of how psychical mechanisms work.

If the assumption that the human psyche contains a non-quantitative dimension is correct, then the replication crisis is not an “accident at work” that happened “just like that” due to unique circumstances. Instead, again speaking with Kuhn and Lakatos, such crises must (almost) inevitably occur for systemic reasons, because the object of investigation, i.e., the human psyche, eludes access to a greater or lesser degree due to the methodology used. This lack of fit between an investigated psychical phenomenon and the method used to investigate it in turn means that unexplained factors exert an influence and thus an explanatory gap exists that cannot be closed by normal science, to use Kuhn’s terminology.

So, if this interpretation of the replication crisis is correct, there are two reciprocal possibilities for the future: First, if the non-quantitative dimension of the human psyche continues to be (largely) neglected, the replication crisis will continue or repeat itself in a similar form because the or at least one of its root causes has not been addressed. Second, if the non-quantitative dimension of the human psyche is considered more intensively, the replication crisis will be mitigated or will not recur in this form, precisely because the or at least one of its root causes has been sufficiently addressed.

The replication crisis could therefore be a symptom that psychology systematically neglects certain basic ontic conditions of its object of investigation, i.e., the human psyche, or only considers them inadequately. And according to Kuhn and Lakatos, such fundamental problems usually lead to profound changes in a scientific discipline, meaning that it is possible that the replication crisis represents the initial stage of such a model crisis.

The arguments discussed in the present study, which, starting from the epistemological status of replications, lead to fundamental philosophical questions, showing that the replication crisis offers an opportunity to ask fundamental questions about the nature of the psyche. In this sense, the replication crisis is not only a problem that challenges the functioning of the discipline but also an opportunity to clarify the foundations of the discipline and to advance the discipline as a whole by improving its access to the human psyche as its object of study.

Karl Popper, Thomas Kuhn and Imre Lakatos, three classics of the philosophy of science, were used to interpret the replication crisis. Finally, a fourth important philosopher of science, Paul Feyerabend, can be used to illustrate another fundamental aspect: The key message in *Against Method* (Feyerabend, 1975) is that the limiting of methodological approaches restricts access to phenomena and thus hinders scientific progress. According to Feyerabend, methodological approaches and frameworks are not only justified by “rational” reasons but reflect a more comprehensive understanding of the world. Ancient Babylonian science, for example, forms a system that is only partially understandable today because it was embedded in a completely different world view. The same applies to Aristotelian science, whose basic assumptions differ fundamentally from today’s science. According to Feyerabend, there are no objective criteria that can rationally justify the superiority of one of these systems. This assumption may or may not be true, but it demonstrates the need to reflect on the general foundations on which science is based because, as the replication crisis suggests, they determine to a large extent how a discipline functions.

However, the results of this study for psychology as a discipline show a peculiarity that has so far received little attention in philosophy and history of science: The falsification of theories, a model crisis, or the degeneration of a research program usually take place at the local level of theories and their concrete content, which relate to specific phenomena. In contrast, this study argues that a very global aspect such as a quantitatively dominated method can be explained by the same mechanisms and can lead to the same situations. It may therefore be that psychology is a special case that differs significantly from other disciplines. It is possible, for example, that all psychological theories that could not be supported by replications are correct in terms of content but that they are not (fully) accessible with a quantitative methodology. Psychology thus represents an interesting case for the history and theory of science, the further investigation of which could not only advance psychology as a discipline but also provide new insights for the history and theory of science.

Returning to psychology itself and the human psyche, the final question that remains is what the above means in concrete terms for psychology as an academic discipline: There are various suggestions as to how psychology could increase its explanatory power by expanding its range of methods (e.g., Hutmacher and Mayrhofer, 2023; Malich and Rehmann-Sutter, 2022; Wiggins and Christopherson, 2019; Juarrero, 2000). This fits in with Feyerabend’s (1975) call not to let the method dominate the research. At the same time, however, the question arises as to whether the possible existence of a non-quantitative dimension in the human psyche does require a different kind of theory that takes this circumstance (better) into account, even if it is not possible to say in advance what this kind of theory should look like.

This study thus suggests that it may be necessary to fundamentally rethink and expand the current framework within which much of psychology operates in order to reflect the full richness of human experience—or, in other words, that the replication crisis started as an epistemological crisis but heralds a model crisis and possibly a paradigm shift. Such a paradigm shift in response to a fundamental problem also involves a different, new way of thinking, the emergence of an entirely different form of theorizing, and the need to develop new concepts that reflect this changed way of thinking—in short, a different *Weltanschauung* concerning the nature of the psyche.

## Author contributions

RM: Writing – original draft, Writing – review & editing, Conceptualization. IB: Writing – review & editing. JH: Writing – review & editing.

## Footnotes

^1^The terms “paradigm” and “model” are usually employed interchangeably. However, the phases in Kuhn’s model are commonly referred to as “pre-science,” “normal science,” “model drift,” “model crisis,” “model revolution,” and “paradigm shift,” with “*paradigm* shift” being used instead of “*model* shift.”

^2^As suggested by Uher (2021), the term “psychical” is used here as adjective for phenomena that relate to the psyche itself, e.g., motivational, cognitive or emotional mechanisms. In contrast, “psychological” is used for research into psychical phenomena, i.e., experiments and other studies or theories on, e.g., motivational or emotional phenomena.
